# Combinations using checkpoint blockade to overcome resistance

**DOI:** 10.3332/ecancer.2020.1148

**Published:** 2020-12-03

**Authors:** Stefania Morganti, Giuseppe Curigliano

**Affiliations:** 1 Division of Early Drug Development for Innovative Therapies, European Institute of Oncology (IEO), IRCCS, Via Ripamonti n.435, 20141, Milan, Italy; 2 Department of Oncology and Hemato-Oncology, University of Milan, Via Festa del Perdono n. 7, 20122 Milan, Italy

**Keywords:** chemo-immunotherapy, immune-checkpoint inhibitors, immunotherapy combinations, immunotherapy resistance, next-generation immune modulators, NGIM, targeted therapy

## Abstract

The advent of immunotherapy for cancer represented a paradigm shift in the treatment approach of neoplasia. Immune-checkpoint inhibitors (ICIs) were demonstrated to significantly improve outcomes, including overall survival across several cancer types, with yearly-durable responses. Nevertheless, many patients derive minor or no benefit with immune checkpoint (IC)-blockade, including patients with cancer types traditionally considered immunogenic. Combination strategies of ICIs with chemotherapy, radiotherapy, targeted therapies or other immunotherapy compounds have been conceived in order to boost the immune-responses and potentially overcome resistance to ICIs. This review focuses on mechanisms underlying resistance to IC-blockade and provides an overview of potential advantages and limitations of combination strategies currently under investigation.

## Introduction

Cancer is a genetic disease, resulting from a multistep tumorigenesis with the selection for clonal malignant cells capable of uncontrolled proliferation, survival, invasion and metastatic potential [1]. Genomic alterations responsible for malignant transformation frequently occur in somatic cells; however, the environmental and spontaneous mutagenesis and expansion of malignant clones are actively supervised by the immune system, able to tackle cancer development and spreading by recognizing and killing individual aberrant cells.

The interaction between cancer cells and the immune system has been elegantly described as a self-propagating cyclic process by Chen and Mellman [2]. This process starts with the release of cancer cell antigens and ends with the killing of tumour cells. Acquisition of immune-stimulating factors occurs during the cycle, leading to anti-tumour T-cell response amplification. On the other hand, the onset of inhibitory signals can generate negative regulatory feedback mechanisms and halt the development or limit the immune response.

The balance between immune-stimulating and immune-suppressing factors drives the immune response against cancer. This dynamic relation has been defined ‘immunoediting’ [3] and is described as a three-phase process: elimination (immune surveillance with clearance of the malignant clones), equilibrium (tumour control with no radical clearance of the clones) and escape (immune-system evasion and tumour progression). Progression from the first two phases to the last one is marked by acquisition of new mutations that allow malignant cells to evade immune-recognition and start uncontrolled proliferation.

Diverse escape mechanisms [3] harbouring immunosuppressive functions have been described. *Inter alia*, the activation of immune-checkpoints (ICs) like cytotoxic T lymphocyte-associated protein-4 (CTLA-4) or programmed cell death protein-1 (PD-1) represents one of the best characterised mechanisms of immune escape, with therapeutic implications. The development of drugs targeting the ICs, like the class of IC inhibitors (ICIs), marked the history of cancer treatment, achieving results never seen in terms of durable responses and long survival across several cancer histologies. Albeit largely effective in a subset of long-responding patients with selected histologies, many cancer types seem to be insensitive to these agents, while others develop resistance.

The aim of this review is to describe main combination strategies currently under development to overcome resistance to ICIs.

## ICIs: mechanism of action and resistance

ICIs are monoclonal antibodies (mAbs) targeting checkpoint proteins expressed by immune cells or tumour cells, such as PD-1, programmed cell death protein-ligand 1 (PD-L1) and CTLA-4. These proteins are physiologically expressed by immune-competent cells to maintain immunological homeostasis and prevent autoimmunity [4, 5], but their upregulation is a well-characterized mechanism of tumour immune-escape [6]. ICIs are able to reactivate T-cell mediated immune response by interrupting co-inhibitory signalling pathways and allowing the killing of tumour cells [7, 8].

The pharmaceutical landscape of development of ICIs is complex, with the recent approval of a multitude of ICIs used as single agents, in combination with other ICIs or with standard treatments. Additionally, the use of pembrolizumab has been Food and Drug Administration-approved for treatment of all tumour types, regardless of the original site of origin and histology, if harbouring microsatellite instability-high (MSI-h) tumours. This approval by the food and drug administration (FDA) has represented the first tissue/site-agnostic approval in the oncology field [9].

Approved ICI combinations are presented in Table 1.

The introduction of ICI-based therapy in the clinical setting has revolutionized the history of oncology, leading to long-term responses and improving survival in a wide range of cancer types. Nevertheless, only 10%–30% of all treated patients derive meaningful clinical benefit from immunotherapy, and only a small subset of them truly achieves long-term survival outcomes. Moreover, many tumours show primary or acquired resistance to ICIs.

In particular, primary resistance occurs when patients progress rapidly and/or do not benefit at all from exposure to immune-regulating agents, whereas secondary resistance refers to cases of relapse and progress after an initial response [10].

**Figure 1.**
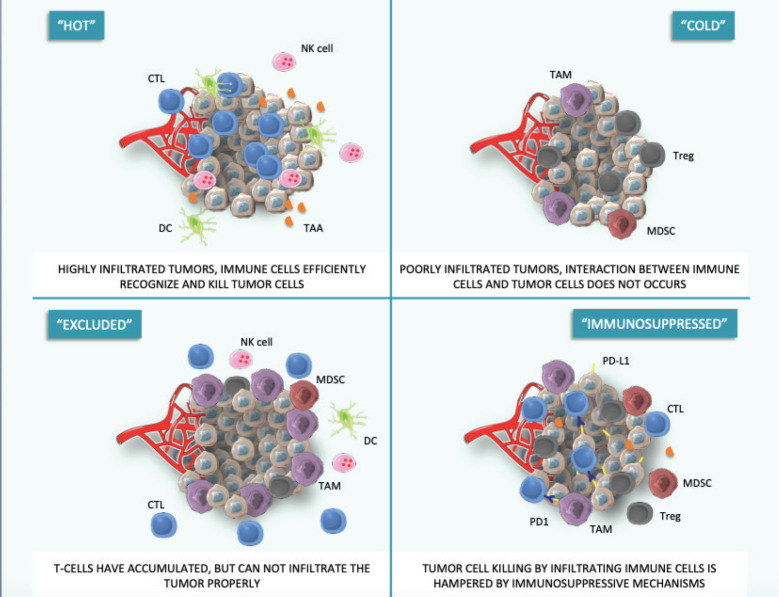
Hot, cold and adapted tumour phenotypes. This figure was created using Servier Medical Art templates, which are licensed under a Creative Commons Attribution 3.0 Unported License; https://smart.servier.com.

The principal causes of primary resistance to ICIs include lack of effective tumour-related antigens, needed for T-cell recognition of the altered self-material, and absence of infiltrating T cells, in a way that interactions between the immune system and cancer cells cannot occur. These cancers are commonly called ‘cold’ tumours, to distinguish them from effectively immune-infiltrated ‘hot’ tumours. In the continuum between ‘cold’ and ‘hot’ tumours, an intermediate phenotype called ‘altered’ has been proposed, in which immune cells are unable to infiltrate the tumour properly (‘excluded’ phenotype) or the immunosuppressive environment limits effector actions of T-cells (‘immunosuppressed’ phenotype) [11] (Figure 1).

In this context, the term ‘adaptive resistance’ refers to all the situations in which cancer cells can be potentially recognised by infiltrating T-cells, but other mechanisms concur to build an immune-suppressive milieu [10]. Adaptive resistance can occur *ab initio* or develop during treatment with IC-blockade, as a form of acquired resistance. Some well-characterized mechanisms of adaptive resistance have been reported, such as loss of tumour antigen [12]- or human leukocyte antigen (HLA) [13]- expression, constitutive expression of co-inhibitory proteins [14], alterations of intracellular signalling pathways (Mitogen-Activated Protein Kinase (MAPK) [15], Phosphoinositide 3-Kinase (PI3K) [16], WNT/β-catenin [17]) and anti-inflammatory cytokines secretion by immune suppressive cell populations (regulatory T-cells [18], myeloid derived stem cells [19], type II macrophages [20]) in the tumour microenvironment (TME). While some of these mechanisms are prevalent in certain tumour types, for the majority of immune-resistant tumours multiple causes overlap and act synergistically.

In order to reverse these mechanisms and overcome resistance to ICI monotherapy, new combination strategies have been explored, integrating ICIs with different agents such as radiotherapy, chemotherapy, targeted agents or new-generation immune-modulators.

## Chemotherapy plus ICIs

Chemotherapy (CT) comprises a large group of drugs with different mechanisms of action and targets. Historically, preclinical trials testing CT agents did not consider the potential interactions between CT and the immune system, and these drugs have been mainly developed *in vitro* and through immunodeficient mice. Similarly, early phase trials in human have broadly analysed the adverse effects on immune-competent cells, such as on the white blood cells count, missing the pharmacodynamics of CT–host immune system interactions. A stronger interest regarding the potential immunomodulatory effect of CT has emerged quite recently, following the ‘immunotherapy revolution’ of last years. CT can both increase immunogenicity of tumour cells and inhibit immunosuppressive features induced in the TME.

Firstly, some CT agents demonstrated to enhance tumour-infiltration, expansion and activity of effector cells like cytotoxic T-lymphocytes (CTLs), dendritic cells (DCs) and natural killer (NK) cells, exerting immune-modulating properties. Cyclophosphamide [21], gemcitabine [22], platinum compounds [23], taxanes [24] and methotrexate 25] have all demonstrated to recruit DCs in the TME, induce their maturation and increase antigen presentation. Treatment with taxanes [26] and cyclophosphamide [27] also leads to increased NK cells activity. Moreover, 5-fluorouracil [28], taxanes [29] and cisplatin [30] have been found to increase the concentration of tumour-infiltrating lymphocytes (TILs), suggesting to facilitate the recruitment of immune-competent cells with a role in mounting an anti-cancer response.

Cancer cells can escape immune-surveillance by inducing a TME dominated by immune-suppressive cells, like regulatory T-cells (Tregs), myeloid-derived suppressor cells (MDSCs) and M2-polarized macrophages.

CT has the potential to deplete the immune-suppressive cell populations. Cisplatin [30], cyclophosphamide [31], taxanes [32], gemcitabine [33], anthracyclines [34] reduce Tregs infiltration and functionality. An augmented CTLs/Tregs ratio has been observed after administration of taxanes [35] and oxaliplatin [36]. Vincristine and dacarbazine suppress tumour-associated macrophages [37]. Finally, taxanes [38], 5-fluorouracil [39], gemcitabine [40] and cisplatin [30] have been demonstrated to deplete the tumour from MDSCs.

Apart from actions on immune cells, CT can stimulate immunogenicity of tumour cells by increasing tumour-associated antigens production, release and presentation to immune-component cells. Immunogenic cell death (ICD) is a form of cell death induced by CT and RT, characterized by secretion of damage-associated molecular pattern proteins, which in turn trigger antitumour immunity by recruiting DCs into the tumour bed and stimulating tumour antigens up-taking, processing and presentation to T cells. Anthracyclines [41], cyclophosphamide [21, 27] and oxaliplatin [42] are powerful inducer of ICD.

Tumour cells can suppress the antigen presentation and T-cell recognition by losing major histocompatibility complex (MHC) I expression on cell surface, a known mechanism of immune escape. Another way to increase tumour immunogenicity shared by several CT drugs is restoration of MHC I expression [43, 44].

Following these evidences, several studies tested CT in combination with ICIs and, as expected, their co-administration was found to act synergistically to induce tumour cell killing and durable responses. Chemo-immunotherapy regimens have shown superiority to first-line CT in several cancer types, with manageable toxicities [45–50]. Table 1 reports all chemo-immunotherapy approved regimens with corresponding indications.

Interestingly, immune-modulating action of CT seems to be related to both dose and schedule of administration. Two *in vivo* studies showed how synergistic effects are maintained only when CT is administered before ICIs or concomitantly, whereas they are lost if administered after it [51, 52]. An induction phase seems to be necessary to maximise tumour killing. Regarding optimal dose of CT, some clinical evidences showed an increased efficacy for low-dose regimens, namely metronomic regimens, boosting the immune system without inducing myelosuppression [53].

The phase II TONIC trial, conducted in the metastatic setting of triple-negative breast cancer, investigated the potential role of short-term ‘induction’ or ‘priming’ with CT or radiation in modulating the anticancer immune response to ICIs. The induction phase consisted in 2 weeks of low-dose CT administration (cisplatin, doxorubicin or cyclophosphamide), RT versus no treatment. Response rates were higher in the cohorts treated with low-dose CT, compared with nivolumab alone, suggesting an immune-enhancing activity. Biomarker analysis showed also a higher number of infiltrating CTLs [54].

CT induction strategies aiming at transforming ‘cold’ into ‘hot’ tumours to potentiate their sensitivity to ICIs are currently being tested in several tumour types. For instance, in the ARETHUSA [55] (NCT03519412) and MAYA (NCT03832621) trials, patients with MGMT-silenced microsatellite-stable (MSS) metastatic colorectal cancers (mCRCs) receive an induction treatment with single-agent temozolomide (TMZ), followed in the absence of disease progression by the combination of TMZ with CTLA-4 plus PD-1 blockade (MAYA trial) or by pembrolizumab at the time of tumour mutational burden (TMB) high-associated disease progression (ARETHUSA trial). While MSI-high mCRCs are sensitive to ICIs blockade, MSS mCRCs usually are not. The investigators hypothesized that induction treatment of MSS-MGMT hypermethylated mCRCs with alkylating agents like TMZ could alter the tumour genetic landscape by increasing TMB, leading to potential sensitisation to ICIs blockade [56].

## Radiotherapy plus ICIs

Radiotherapy (RT) has many important indications in cancer treatment, both in the curative and palliative setting. For the metastatic diseases, the aim of RT is to improve local control and relieve cancer-related symptoms like pain or bleeding. After the advent of immunotherapy, a renewed interest emerged in the potential role of RT in modulating the immune system.

Whereas RT has been historically considered mainly suppressive because of deep lymphodepletion in irradiated sites, recent studies showed instead a more complex interaction.

RT causes death of both tumour and immune cells. A relative increase in locally suppressive immune-cell is recorded immediately after RT, because of their lower immune-sensitivity. Despite this, a rapid repopulation by other lymphocyte subsets occurs, with recruitment of circulating effector immune cells. A phenotype change in tumour cells survived to RT also occurs, with an increased recognition by surrounding T-cells [57]. Finally, RT induces local secretion of inflammatory cytokines [58] by immune cells, increasing immune sensitisation further.

Besides local effects, RT can trigger immune killing outside the irradiated site, providing a so-called ‘abscopal effect’: this phenomenon, firstly described by R. H. Mole in 1953, consists on systemic responses induced by RT but in distant sites not irradiated [59]. A potential correlation between the abscopal effects and an immune stimulation was firstly proposed in 2004, after the observation that this phenomenon does not occur in T cell-deficient mice [60]. The abscopal effect can be assimilated to an ‘in situ vaccination’: RT induces an immunogenic-cell death, with the subsequent release of immunogenic factors. These molecules then trigger an antigen-specific immune response at a systemic level, beyond the sites of irradiation.

In recent years, several clinical experiences combining RT and IC-blockade have further corroborated the relation between the abscopal effect and a systemic immune activation. Formenti *et al* [61] enrolled patients with metastatic chemo-refractory NSCLC and reported a higher response rate in patients who received RT plus ipilimumab than ipilimumab alone or with CT. In head and neck squamous cell carcinoma (HNSCC) response rate was also higher when pembrolizumab was combined with RT than administered alone, as emerged from the PEMBRO-RT study [62]. However, a similar trial testing nivolumab plus RT versus nivolumab alone in the same tumour type did not recorded any advantage in terms of responses or progression-free survival from the combination therapy [62].

In addition, the PACIFIC trial showed a statistically significant and clinically meaningful benefit from the addition of maintenance durvalumab after chemo-radiation for locally advanced NSCLC [63].

CT and RT act synergistically to increase immune recognition of tumour cells, by inducing an ICD, increasing antigen recognition and reducing activity of immune-suppressive cells. However, PD-L1 is usually upregulated by these treatments, hampering action of T-cell. Administration of ICIs after CT-RT removes this inhibition, rearming effector cells against tumour cells.

Doses, time and fractioning can also influence the immune effect of RT. Some preclinical evidences showed higher concentration of tumour-infiltrating T cells after single dose [64, 65] than in conventional fractionated low-dose regimens. However, other studies proved the opposite, with better results achieved with fractionated RT [66, 67]. With regard to timing of RT administration, a shorter interval between radiation and ICI administration seems to increase efficacy. In the PACIFIC trial, better outcomes were recorded when durvalumab was initiated within ≤2 weeks of RT rather than >2 weeks after [63].

These evidences teach us that RT can potentially improve efficacy of IC-blockade, both increasing tumour immunogenicity and triggering the immune system. Nevertheless, more studies are needed to understand which is the best strategy to adopt.

## Targeted agents plus ICIs

Similarly to what previously exposed for CT and RT, also targeted agents interact with the immune system.

Targeted agents are mainly monoclonal antibodies (mAb), able to induce an immune response because of their intrinsic nature independently from the target. By binding both immune cells (through the Fc-gamma receptor) and tumour cells, mAb can induce the so-called antibody-dependent cellular cytotoxicity (ADCC) reaction, by which the release of perforin and granzyme induces an immune-mediated tumour cell killing [68, 69]. Specific polymorphisms of Fc-gamma receptor have been associated to improved mAb effector functions [70].

Monoclonal antibodies can also stimulate complement-dependent cytotoxicity: the complement proteins can bind the Fc region of the mAb, inducing the assembly of the membrane attack complex and finally the tumour cell lysis [71].

An increased expression of proteins involved in tumour antigen processing has also been associated to targeted therapies. For instance, cetuximab therapy was shown to increase expression of transporter associated with antigen processing (TAP)1/2 [72], whereas vemurafenib demonstrated to induce calnexin and calreticulin proteins [73], all involved in antigens processing. Moreover the same drugs were associated to increased expression of MHC class I and II [74, 75]. Also the qualitative pool of tumour-associated antigens presented was found to be altered by targeted drugs, in a way that makes tumour cells more recognizable by effector cells [76].

Regarding PDL1 expression, some evidences demonstrated a reduced expression when these agents were administered, whereas others found the opposite [77, 78].

Finally, targeted agents were shown to influence the immune infiltrate, both depleting immunosuppressive cells [79] and recruiting effector immune cells [80], as well as eliciting immunogenic cell death [81].

A synergistic effect induced by the combination of ICIs and TT has been demonstrated in several preclinical experiences [82, 83], leading to the design of clinical trials aimed at investigating the outcome of this synergism in terms of efficacy and tolerability.

An initial phase I trial tested the combination of ipilimumab and vemurafenib in patients with metastatic BRAF-mutated melanoma [84], but it was stopped early because of excessive toxicity. Other studies combining BRAF-inhibitors (BRAFi) and ipilimumab with or without MEK-inhibitors (MEKi) showed similar results in terms of toxicity, with a high rate of G3-4 colitis and hepatic toxicity [85, 86]. Combinations of BRAFi, MEKi and anti-PD(L)1 were instead better tolerated, with promising results in terms of response rate [87]. Two different triple combined therapies are currently under investigation by two phase III randomized trials (COMBI-I—NCT02967692; and TRILOGY IMspire150—NCT02908672).

As previously reported for CT and RT, also in these trials the sequence of administration between TT and IC-blockade seemed to impact on both efficacy and toxicity.

Non-squamous NSCLC is a clinical model of oncogenic-addicted cancer, for which gene alterations driving the tumorigenesis are well characterized and molecular drugs targeting their products are widely used in clinical practice. While targeted therapy has impressively improved outcomes in these diseases [88–90], immunotherapy seems to be not effective in the majority of patients with oncogene-addicted NSCLC [91]. Genomic alterations found in NSCLC have been mostly associated to lower mutational load and immunogenicity [92–94], with poorer response rates observed for patients harbouring these mutations in all clinical trials using ICIs.

Epidermal growth factor receptor (EGFR)-mutated NSCLC are essentially ‘immune-excluded’ cancers [94]. EGFR-mutation can induce PDL1 expression [95], but the very low rate of TILs along with the low number of neo-antigens makes IC-blockade ineffective [96, 97]. Similarly, the combination of anti-EGFR tyrosine kinase inhibitor (TKI) plus ICIs does not significantly improve activity versus targeted therapy alone, with instead a more severe toxicity [98–100].

ALK-rearranged NSCLCs have a similar immune-excluded phenotype and do not benefit from ICI treatment [96]. However, preclinical studies showed that ALK inhibitors (ALKi) could enhance T-cell infiltration and proliferation, with also an increased cytokine production [101]. Several early phase trials showed a high response rate with the combination of ALKi and ICIs, but at the cost of severe toxicities [102, 103]. The administration of new generation ALKi with ICIs seemed instead to be well tolerated, with promising efficacy data [104].

With the purpose of exploit the off-target immune stimulation of targeted agents, some trials are testing such combinations also in patients otherwise not eligible for these drugs. For instance, an ongoing trial is investigating the combination of durvalumab + monalizumab + cetuximab in RAS mutated metastatic CRCs (NCT02671435). Preclinical data have shown that monalizumab can enhance the ADCC activity of cetuximab; and in turn tumour cell death induced by cetuximab can potentially lead to release of tumour antigens and finally stimulate the antitumour immune response [105].

A similar strategy has been pursued also in BRAF-wt melanoma. A phase III trial (NCT03273153) testing the combination of cobimetinib and atezolizumab versus pembrolizumab alone has been designed, following promising preclinical and phase I data [106] showing an enhanced antitumour immune response to ICIs induced by MAPK inhibition. However, this trial did not meet the primary endpoint of progression-free survival (PFS), and G≥3 adverse events were more common with the combination than with pembrolizumab (67% versus 33%) [107].

## Antiangiogenic drugs plus ICIs

Angiogenesis processes are typically upregulated by tumours in order to provide nutrients and oxygen to proliferating cancer cells [1]. Drugs targeting the vascular endothelial growth factor (VEGF), a key regulator of angiogenesis in cancer, can inhibit this mechanism and reduce tumour growth, with favourable outcomes also in terms of survival in different cancer types [108].

Several preclinical evidences have described a strong interaction between angiogenesis mechanisms and the immune system. VEGF secretion not only blocks T-cell development and infiltration into the tumour, but also induces proliferation of immune-suppressive cells, like MDSCs and Tregs [109, 110]. The administration of antiangiogenic drugs like sunitinib or cabozantinib can reverse this immune-suppression, both increasing intratumoural effector cells, decreasing PDL1 expression and reducing infiltrating MDSCs and Tregs [111–114].

A potential synergism between antiangiogenic drugs and ICIs has been confirmed in preclinical models [115], leading to the development of clinical trials in different cancer types.

In patients with renal cell carcinoma (RCC), safety and efficacy of combined treatment with IC-blockade and antivascular agents have been extensively investigated. Several phase I trials demonstrated the feasibility and promising activity of such combinations, which consequently moved to first-line setting [116–118]. Results from two big phase III trials, the JAVELIN Renal 101 [119] and the KEYNOTE-426 [120], have been recently presented. Both of them used sunitinib as standard arm, whereas axitinib was associated to avelumab or pembrolizumab, respectively, as first-line regimen of treatment. Median PFS was significantly higher in the experimental arm in both trials, whereas median overall survival (OS) is still not reached. The median PFS observed in the pembrolizumab plus axitinib arm (15.2 m) was the highest ever-observed in mRCC. Toxicity was not significantly increased in the combination arm in both trials.

A significant median OS advantage from the combination of atezolizumab plus bevacizumab has been recently reported also in patients with advanced hepatocellular carcinoma [121].

## Combination of different ICIs

CTLA-4 and PD-1/PD-L1 checkpoints act on different phases of immune activation: CTLA-4 regulates T-cell proliferation in the early phases of immune response, primarily in lymph nodes, whereas PD-1/PD-L1 axis acts later, primarily in peripheral tissues or into the TME [122]. These differences provide the rationale for combining anti-PD(L)1 with anti-CTLA4 mAbs, with a supposed additive or synergic action in immune stimulation. Clinical trials testing ipilimumab plus nivolumab firstly showed improved and durable responses in patients with advanced melanoma (ORR 58%, median duration of response NR), with a significantly increased OS (median OS not reached after 60 months of median follow up) [123].

Despite these exciting data, this combination is not universally recognized as standard first-line regimen because of its safety profile. In the CheckMate-067 trial, G3-4 adverse events (AEs) were observed in 55% of patients in the combination arm, versus 16% and 27% of patients treated with nivolumab or ipilimumab monotherapy, respectively. Moreover, evidences about a significant advantage of combination therapy over anti-PD-1 alone are weak, given that the CheckMate-067 trial was not powered to identify differences between these two regimens. Also for other tumour types in which this combination is approved, such as mRCC and MSI-h CRC, this superiority cannot be affirmed: in RCC nivolumab plus ipilimumab was tested versus sunitinib [124], and in MSI-h CRCs no comparison arms were present [125].

‘Next-generation immune-modulators’ (NGIMs) are antibodies targeting signalling pathways other than PD-(L)1 and CTLA4, involved in immune stimulation or suppression. Several co-stimulatory and co-inhibitory molecules with a recognized role in the interaction between tumour and the immune system have been characterized, and many of these pathways have been linked to adaptive resistance to IC-blockade [126]. These drugs are commonly also defined ‘second-generation ICIs’, but because of their action extended beyond checkpoint-inhibition the term ‘immune modulation’ should be preferred. Despite recent progresses, the understanding of complex biology underlying these pathways is still far from complete, and for most of them the identification of all associated ligands/receptors and the exact mechanism of action remain unresolved.

All of these molecules can be classified on the basis of their primary action, as co-stimulatory or co-inhibitory, and distinguishing cells in which they are predominantly expressed: lymphoid cells, NK cells or non-lymphoid cells (innate immune system, tumour cells or cells in the TME) [126]. Some of them are expressed on T-cell surface and act similarly to checkpoint inhibitors, like T cell immunoglobulin and mucin domain-containing protein 3 (TIM3), Lymphocyte-activation gene 3 (LAG3), T-cell immunoglobulin and ITIM domain (TIGIT) and V-domain Ig suppressor of T cell activation (VISTA) [127]. Other molecules act instead modulating the TME, regulating chemokine secretion (CXCR4, CXCL12) [128], concentration of metabolic products (Indoleamine 2,3-dioxygenase 1 (IDO1) [129] or activity of innate effectors or immune-suppressive cells (NGK2A [130], Colony stimulating factor 1 receptor (CSF1R) [131]).

NGIMs are currently tested in combination with anti-PD(L)1 mAb, in response to preclinical evidences showing a potential synergic action. The combination of nivolumab and anti-LAG3 relatlimab is under investigation in several cancer types, with preliminary data showing encouraging efficacy in patients with melanoma progressing on previous ICIs [132]. Similarly, treatment with MBG453, an anti-TIM3 mAb, and spartalizumab showed good tolerability and preliminary signs of anti-tumour activity in a phase I trial enrolling all solid tumours [133]. Unfortunately these data were not confirmed in the phase II study testing the same combination in melanoma and NSCLCs progressed on PD-(L)1 therapy [134]. Other anti-TIM3 mAb are under investigation (Sym023, TSR-022 and LY3321367).

IDO1 is a rate-limiting enzyme converting tryptophan to kynurenine. High concentrations of kynurenine in the TME has been linked to T-cell anergy and resistance to ICIs [129]. After preclinical and early clinical promising evidences of efficacy, the IDO1-inhibitor epacadostat has been tested in combination with pembrolizumab versus pembrolizumab alone in a large phase II/III trial. This trial failed to demonstrate any advance with the combination therapy [135].

Description of mechanisms of action and available evidences of all of NGIMs under development is beyond the aim of this review, and well-described elsewhere [126].

## Conclusion

Despite impressive results observed with ICIs in several cancer types, still many patients do not achieve responses or eventually develop adaptive resistance. New combination strategies between ICIs and different agents have been conceived in order to overcome this resistance and increase the percentage of patients that can benefit from immunotherapy. Combination of ICIs with CT, RT, targeted agents or NGIMs showed a potential synergistic activity in preclinical series, leading to the design of several clinical trials. As previously reported, some strategies lead to a real clinical benefit, whereas others were disappointing because of inefficacy or excessive toxicity.

Although progresses of recent years shed a bit of light in this complex landscape, there are many open questions that need to be addressed. Optimal doses and timing of administration of such combinations are still uncertain. Some evidences seemed to suggest that both CT and RT could be more immunogenic at lower than standard doses, but no comparative big trials are available.

Combination therapies previously reported demonstrated to increase activity and efficacy, but usually at the cost of higher toxicity. In some settings, immunotherapy alone proved to significantly increase survival outcomes, and the question of whether a combination strategy is needed for all patients has been raised. For instance, both immunotherapy alone and IT-CT combinations are available first-line options for advanced NSCLC with PD-L1 expression higher than 50%, and no comparative trial is available. Subgroups analysis and meta-analysis suggest that women, never smokers, patients with tumour-related symptoms or heavy tumour burden seem to mostly benefit from a combination strategy in this setting [45, 46, 136–138].

Another challenge is about how to combine or sequence ICIs with other agents. In melanoma, the triple combinatorial therapy with BRAFi plus MEKi and ICIs demonstrated to increase anti-tumour activity but with an increased toxicity [87]; therefore, a sequential strategy could be the preferential choice if efficacy is maintained.

Comparisons between different combination strategies are also lacking. In mRCC both the combination of nivolumab plus ipilimumab and axitinib plus anti-PD1 increased outcomes versus sunitinib in the first-line setting, but no direct comparison is available and choices in clinical practice are mainly derived from differences emerged in subgroup analysis.

**Figure 2.**
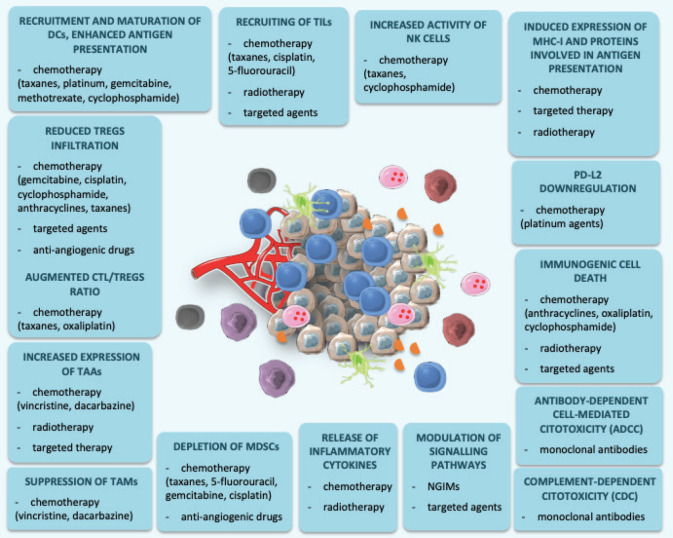
Immunological effects of CT, RT, targeted agents and new-generation immune modulators. This figure was created using Servier Medical Art templates, which are licensed under a Creative Commons Attribution 3.0 Unported License; https://smart.servier.com.

In conclusion, combinations between ICIs and different agents such as CT, RT, target agents or NGIMs demonstrated to increase efficacy of immune response against cancer, and potentially overcome resistance to single-agent immunotherapy (Figure 2). However, several questions are still unanswered, and future research should be directed to identify the best strategy in terms of dose, timing, duration and sequence of administration.

## Funding

This work was partially supported by the Italian Ministry of Health with Ricerca Corrente and 5x1000 funds.

## Figures and Tables

**Table 1.** Approved combination of ICI (September 2019).

Trial	Regimen	Disease and setting	Results
KEYNOTE-189 Gandhi *et al* [45]. Gadgeel *et al* [139].	Pembrolizumab plus pemetrexed and platinum CT	Metastatic non-squamous NSCLC, with no EGFR and anaplastic lymphoma kinase (ALK) genomic alterations^a^ First-line treatment	mPFS 8.8 versus 4.9 months (HR 0.52; 95% CI, 0.43–0.64) mOS 22 versus 10.7 months (HR 0.56; 95% CI 0.45–0.70)
KEYNOTE-407 Paz-Ares *et al* [46].	Pembrolizumab plus carboplatin and paclitaxel or nab-paclitaxel	Metastatic squamous NSCLC^a^ First-line treatment	mOS 15.9 versus 11.3 months (HR 0.64; 95% CI, 0.49–0.85)
IMpower150 Socinski *et al* [47].	Atezolizumab plus bevacizumab, paclitaxel and carboplatin (versus bevacizumab plus paclitaxel plus carboplatin)	Metastatic non-squamous NSCLC, with no EGFR and ALK genomic alterations^a^ First-line treatment	mOS 19.2 versus 14.7 months (HR 0.78; 95% CI, 0.64–0.96)
IMpower130 West *et al* [48]	Atezolizumab plus paclitaxel and carboplatin (versus paclitaxel plus carboplatin)	Metastatic non-squamous NSCLC, with no EGFR and ALK genomic alterations^c^ First-line treatment	mOS 18.6 versus 13.9 months (HR 0.79; 95% CI 0.64–0.98)
IMpower133 Horn *et al* [49]	Atezolizumab plus carboplatin and etoposide (versus carboplatin plus etoposide)	Extensive-stage SCLC^a^ First-line treatment	mOS 12.3 versus 10.3 months (HR 0.70; 95% CI 0.54–0.91)
KEYNOTE-048 Burtness *et al* [50]	Pembrolizumab plus platinum and fluorouracil (versus platinum plus fluorouracil plus cetuximab)	Metastatic or unresectable, recurrent HNSCC^a^ First-line treatment	mOS 13 versus 10.7 months (HR 0.77; 95% CI 0.63–0.93)
IMpassion130 Schmid *et al* [140] Schmid *et al* [141]	Atezolizumab plus nab-paclitaxel (versus nab-paclitaxel)	Metastatic or unresectable TNBC with PD-L1≥1%^a^ First-line treatment	mPFS ITT 7.2 versus 5.5 months (HR 0.8; 95% CI 0.69–0.92) mPFS PDL1+ 7.5 versus 5 months (HR 0.62; 95% CI, 0.49–0.78) mOS ITT 21 versus 18.7 months (HR 0.86, 95% CI 0.72–1.02) mOS PDL1+ 25 versus 18 months (HR 0.71, 95% CI 0.54–0.94)
CheckMate-067 Wolchok *et al* [142] Larkin *et al* [123]	Nivolumab plus ipilimumab (versus nivolumab alone versus ipilimumab alone)	Metastatic or unresectable melanoma^a^	mPFS 11.5 versus 6.9 versus 2.9 months (HR 0.43; 95% CI 0.35–0.52)^d^ mOS NR versus 36.9 versus 19.9 months (HR 0.52; 95% CI 0.42–0.62)^d^
CheckMate-214 Motzer *et al* [124]	Nivolumab plus ipilimumab (versus sunitinib)	Advanced RCC at intermediate or poor risk^a^ First-line treatment	mOS NR versus 26.6 months HR 0.66; 95% CI 0.54–0.80 mPFS 8.2 versus 8.3 months HR 0.77; 95% CI 0.65–0.90 ORR 42% versus 29%
CheckMate-142 Overman *et al* [125]	Nivolumab plus ipilimumab (non randomized)	MSI-H or dMMR metastatic CRC progressed on fluoropyrimidine, oxaliplatin and irinotecan^b^	ORR 55%, mDOR NR 12-months PFS 71%
KEYNOTE-426 Rini *et al* [120]	Pembrolizumab plus axitinib (versus sunitinib)	Advanced RCC^a^ First-line treatment	mPFS 15.1 versus 11.1 months HR 0.69; 95% CI 0.57–0.84
JAVELIN Renal101 Motzer *et al* [119]	Avelumab plus axitinib (versus sunitinib)	Advanced RCC^a^ First-line treatment	mPFS 13.8 versus 8.4 months HR 0.69; 95% CI 0.56–0.84

a Both FDA- and EMA-approved (for HNSCC combination strategy is approved in Europe only for PDL1+ patients)

b Only FDA-approved, under accelerated approval procedure based on ORR and DOR data

c Only EMA-approved

d HR for nivo/ipi versus ipi

mOS: median Overall Survival
